# Breeding parameters on a mink farm infected with Aleutian mink disease virus following the use of methisoprinol

**DOI:** 10.1007/s00705-019-04375-x

**Published:** 2019-08-19

**Authors:** Marek Kowalczyk, Bolesław Gąsiorek, Krzysztof Kostro, Ewa Borzym, Andrzej Jakubczak

**Affiliations:** 1 Department of Biological Basis of Animal Production, Faculty of Biology, Animal Sciences and Bioeconomy, University of Life Sciences in Lublin, Akademicka 13, 20-950 Lublin, Poland; 2 Department of Epizootiology and Clinic of Infectious Diseases, Faculty of Veterinary Medicine, University of Life Sciences, Głęboka 30, 20-612 Lublin, Poland; 3 Department of Fish Diseases, National Veterinary Research Institute, 57 al. Partyzantów, 24-100 Pulawy, Poland

## Abstract

Aleutian mink disease virus is one of the greatest threats to modern mink farming. The disease reduces fecundity and causes high mortality among kits. The aim of this study was to evaluate the effectiveness of methisoprinol in counteracting the effects of Aleutian disease, both by inhibiting replication of the virus and by mitigating the harmful effects of the disease on the fecundity and weight of infected animals. The study included 300 individuals with confirmed infection, divided according to antibody titres into three experimental groups, which received a 20% methisoprinol solution, and three control groups, which did not receive the immunostimulant. In the mink from the experimental groups, the number of copies of the genetic material of the virus in the spleens and lymph nodes was one order of magnitude lower than in the case of the control groups. Mink receiving the supplement also showed higher fecundity (on average 5.83 in the experimental groups and 4.83 in the control groups), and the weight of their offspring before slaughter was over 200 g higher. Given the lack of effective methods for immunoprophylaxis and treatment, methisoprinol supplementation can be an effective means of counteracting the effects of AMDV on persistently infected farms.

**Electronic supplementary material:**

The online version of this article (10.1007/s00705-019-04375-x) contains supplementary material, which is available to authorized users.

## Introduction

Intensive mink farming and breeding are associated with increased exposure of the animals to infectious agents that have a negative impact on their health and well-being. One of the most common diseases affecting mink farming is Aleutian disease (AD), caused by Aleutian mink disease virus (AMDV) – an ssDNA virus belonging to the genus *Amdoparvovirus*, family *Parvoviridae*.

AD is a chronic and incurable disease in mink that results in breeding losses due to frequent abortion, low fecundity, and high juvenile mortality. Low reproductive parameters, manifested as a small number of reared kits, translate directly into the number of pelts produced and the profitability of breeding [1].

AMDV exhibits tropism for immune cells, especially B lymphocytes. The persistent and rapid replication of the virus in the body of infected mink leads to impairment of immune functions. The virus stimulates the host to synthesize excessive quantities of specific antibodies, leading to hypergammaglobulinaemia and the formation of immune complexes. When these complexes are deposited in tissues, they cause inflammation and pathological changes [2]. The disease can have a varied clinical course, from transient infections to persistent and progressive infections [3].

Due to the lack of causative treatment and specific immunoprophylaxis, as well as the growing problem of asymptomatic AMDV infections, there is a need for research on alternative methods to control Aleutian disease and minimize its harmful effects. One such method is nonspecific immunostimulation, which involves enhancing innate immunity using immunomodulatory drugs.

One immunostimulant preparation used against viral diseases is methisoprinol (inosine pranobex or Isoprinosine), a combination of inosine, acetamidobenzoic acid, and dimethylaminoisopropanol. Methisoprinol affects both humoral and cellular mechanisms of the immune response, through the activation of T and B lymphocytes, stimulation of macrophage proliferation and activity, and increased synthesis of interferon [4]. The preparation has been shown to reduce virus titres in cell cultures infected with Aujeszky’s disease virus and Newcastle disease virus [5]. The aim of this research was to evaluate selected breeding parameters on a mink farm persistently infected with AMDV following the use of a 20% solution of methisoprinol, a drug with immunopotentiating properties.

## Materials and methods

### Experimental animals

The research was conducted from November 2015 to the end of October 2016 at a mink farm located in the Wielkopolska Voivodeship, with 10,000 females in the foundation stock. The sanitary condition of the farm was assessed as good. The mink were fed in a traditional system according to recommended standards for this species and for individual periods of the breeding cycle, including mineral and vitamin supplements and permanent access to water. Table 1 presents the ranges of metabolic energy (ME) values and the percentages of ME contributed by individual nutrients in the diet during the annual breeding cycle.

**Table 1:** Demand for metabolic energy in the yearly mink breeding cycle and the percentage share of ME from individual constituents in the feed ration

	Percentage share of ME			
	Protein	Fat	Carbohydrates	ME
Period 1 (1 Dec 2014 – 15 May 2015)	40-50%	30-40%	12-20%	1100-1400 kcal/kg
Period 2 (16 May – 15 July 2015)	40-45%	35-45%	12-20%	1200-1500 kcal/kg
Period 3 (16 July –15 Sept 2015)	33-40%	42-50%	15-20%	1500-1800 kcal/kg
Period 4 (16 Sept 2015 until slaughter)	30-35%	40-50%	15-25%	1400-1800 kcal/kg

AMDV infections are endemic on the mink farm under study in the experiment. They have been confirmed by counter-current electrophoresis, ELISA assays, and PCR. A total of 300 female brown mink with positive ELISA results were selected for the experiment.

The biological material for serological diagnosis of AMDV comprised blood samples collected from live mink before the start of the experiment by toenail clipping into capillary tubes. The samples were tested using ELISA assays (Scintilla Development Company LLC, ADV Antibody ELISA).

The mink were initially divided into three experimental groups (groups 1, 2 and 3) and three control groups (groups C1, C2 and C3) of 50 animals each according to the antibody titres obtained in the ELISA assay. Group 1 and control group C1 comprised minks in which the ELISA assay demonstrated an antibody titre designated as + according to the recommendations of the ELISA assay manufacturer; groups 2 and C2 were mink with a ++ antibody titre; and groups 3 and C3 were animals in which the antibody titre was designated as +++. The research was carried out with the consent of the Second Local Ethics Committee for Experiments on Animals at the University of Life Sciences in Lublin (resolution no. 30/2010).

We used a 20% solution of methisoprinol (VetAgro) for active nonspecific immunostimulation of the mink. The choice of this immunostimulant is justified both by the results of preliminary tests and literature reports confirming its antiviral and immunostimulatory effect [4–9].

In the first stage of the experiment, i.e., from the preparatory period to the end of whelping, 20% methisoprinol was administered only to females of the foundation stock in the experimental groups (groups 1, 2 and 3, with different baseline antibody titres), twice a month with a three-day interval in December, January and February, and additionally twice with a three-day interval in the second half of pregnancy (day 35 – completed organogenesis period), at an oral dose of 40 mg/kg body weight. The mink from the control groups (C1, C2 andC3) received the same feed as the experimental animals but without methisoprinol.

In the second stage of the experiment (after whelping was completed), the first dose of 20% methisoprinol was administered to females of the foundation stock in the experimental groups together with their four-week-old offspring (first transition to solid feed) and then again at the age of eight weeks (weaning period), at an oral dose of 40 mg/kg body weight. Thereafter, both the females in the experimental groups and their juvenile offspring received an oral dose of the preparation in the amount of 40 mg/kg body weight, twice a month with a three-day interval until slaughter, i.e., until the end of October.

Methisoprinol was mixed with the feed ration. An automatic feed system was used, which facilitates uniform feed administration to each cage. The immunostimulant was added to each portion (for each cage) individually.

Reproductive parameters and the body weight of the offspring were assessed in the groups of experimental and control females. Reproductive parameters of females in all groups were assessed on the 10th day after whelping and at the end of the breeding period. The body weight of the offspring was assessed after the end of the breeding period (November 15).

### DNA extraction

DNA was extracted from the spleen and lymph nodes, which were cut into small pieces weighing 5 mg. DNA was isolated using a commercial DNeasy Blood and Tissue Kit (QIAGEN) according to the manufacturer’s protocol with some modifications. Tissues were lysed using Tissuelyser II (QIAGEN) for 20 seconds at a frequency of 20 1/s, and then 200 μl of ATL buffer was added. Samples were washed twice with AW2 buffer. The elution was prepared in a 150-μl volume.

### Quantitative assessment of the numbers of copies of AMDV by qPCR

A total of 84 mink were tested, including 21 females from the foundation stock (three experimental groups) and 21 of their offspring, which had received an oral dose of 20% methisoprinol in the amount of 40 mg/kg BW according to the design described above. The remaining 42 minks (21 females from the foundation stock together with their offspring), which did not receive 20% methisoprinol, were used as controls. After slaughter, lymph nodes (n = 168) and spleens (n = 84) were taken from the mink of both groups, and DNA was extracted from them – 84 samples from the lymph nodes and 84 from the spleens.

Viral load in the spleen and lymph nodes was determined using a qPCR assay targeting a conserved sequence of the NS1 gene. To determine the number of copies of the genetic material of the virus, qPCR was performed using primer 1 (5’- GAAGAATACTGGCAACTCACAACCT-3’), hybridizing to nucleotides 253–277, and primer 2 (5’- GTCGCAGTTTTCCGTGTTCA-3’), hybridizing to nucleotides 303–322 of AMDV isolate HY F17 NS1 (accession number KM374812.1), a TaqMan probe (5’-FAM- CAAAGAGTGCAGAAAGT-BHQ1-3’), and an 88-bp nucleotide construct serving as a size standard in the qPCR reaction. The 88-bp recombinant plasmid construct was obtained from a commercial company Genomed S.A. (Warsaw), and serial dilutions of a stock solution containing recombinant plasmid DNA (AMDV, 10^6^ to 10^0^ copies/μL) were used to create a standard curve.

All samples were initially tested by real-time PCR using a commercial kit (QuantiTectprobe PCR Kit) and a Rotor-Gene Q thermal cycler (QIAGEN, Germany). Assays were performed in a 20-µl reaction volume consisting of 400 nM of each primer and probe. Assays were performed using a CFX Connect Real-Time System (Bio-Rad, USA). The samples were initially kept at 50 °C for 2 min followed by 10 min at 95 °C, and then 50 temperature cycles of 15 seconds at 95 °C and 1 minute at 55 °C.

Serial dilutions of a stock solution containing recombinant plasmid DNA (AMDV, 10^6^ to 10^0^ copies/μL) were used to construct a standard curve for quantification of DNA. To transform Cq values obtained in the qPCR assays to the number of viral copies, we used the following formula: Number of copies = 10^((Cq-b)/m)^, where m is the slope and b is the intercept from the regression equation. Negative and positive controls were included in each reaction run.

### Statistical analysis

The results of quantitative analysis of viral load and breeding parameters were analysed by analysis of variance (ANOVA) to determine whether there were any statistically significant differences between the means. The calculations were made using the SAS statistics package (SAS Institute, Cary, NC, USA). Statistical significance was established as *p* ≤ 0.05.

### Amplification, sequencing and bioinformatic analysis

DNA extracted from spleens (n = 84) was used for PCR amplification with two pairs of primers targeting the VP2 structural protein fragment [10]. The primer sequences, reaction conditions, and composition of the mix are given in Tables 2 and 3. The PCR products were separated in a 1% agarose gel with ethidium bromide at 60 V. The reaction products were subsequently used in the sequencing reaction.

**Table 2:** Primer characteristics and annealing temperature

Name of primer		Primer sequence (5’-3’)	Product length	Annealing temperature	Reference
RP2	Forward	TCTAGAAGCAACGCTTGGGGTGTATG	802 bp	58°C	Costello et al. 1999
	Reverse	GTTGTGTCACTCCACTGTCT			
RP3	Forward	TCTAGATTGGGCCTACCTCCTCTCTG	681 bp	58 °C	
	Reverse	ATACAGGACCAACGTTGTCT

**Table 3:** Reaction mix composition and temperature profiles for each primer pair

Reaction mix	RP2	RP3
Water	to 25 µl	to 25 µl
Buffer	0.97x concentrated	1x concentrated
GC Enhancer	0.05 in final concentration	0.04 in final concentration
Mg2^+^	2.5 mM	2.5 mM
dNTP	0.8 mM	0.8 mM
Forward	1.2 μM	0.8 μM
Reverse	1.2 μM	0.8 μM
Polymerase	1.43 U	1.5 U

Cycling conditions	Temperature	Time	Temperature	Time
Initial denaturation	95 °C	10 min	95 °C	10 min
Denaturation	95 °C	30 s	95 °C	30 s
Annealing	58 °C	45 s	58 °C	45 s
Elongation	72 °C	60 s	72 °C	60 s
Final elongation	72 °C	10 min	72 °C	10 min

PCR products for the V2 primers were purified using an ExoSAP-IT kit (Affymetrix). The same primers as for the original amplification were used for sequencing PCR. A BigDye® Terminator v. 3.1 Cycle Sequencing Kit (Applied Biosystems) was used, and the reactions were carried out according to the manufacturer’s instructions. The sequencing PCR products were purified using a DyeEx Spin Kit (QIAGEN) on a QIAcube apparatus. The samples were subjected to thermal denaturation in formamide. The sequencing reaction for the forward and reverse primers was carried out in a 3100-Avant Genetic Analyser (Applied Biosystems). Sequencing results were analysed using Baser DNA software. The sequences used in the alignment were taken from the NCBI bioinformatic database. Sequence editing and alignment were carried out using MEGA6 and Bioedit software.

## Results

### Results of quantitative AMDV assessment in the submandibular lymph nodes and spleen

**Fig. 1:**
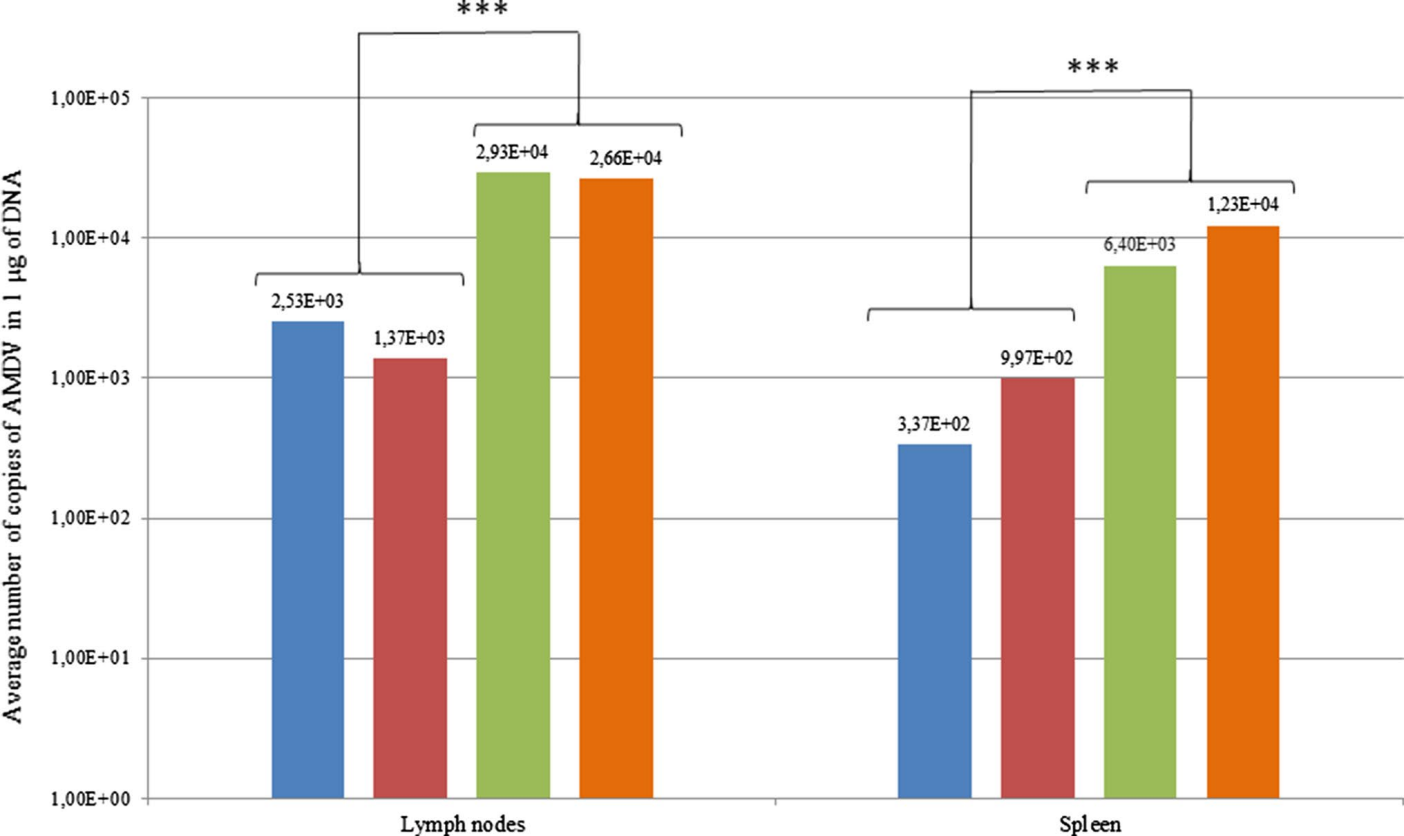
Average number of copies of AMDV DNA in the spleens and lymph nodes of mink in the experimental and control groups in 1 μg of DNA. Blue, experimental group, young female offspring; red, experimental group, adult females from foundation stock; green, control, young female offspring; orange, control, adult females from foundation stock; ***, statistically significant difference

The experimental groups consisting of young and adult females showed a viral titre one order of magnitude lower than in the controls in both the spleen and the lymph nodes (statistically significant difference, *p* = 0.05) (Fig. 1). The average number of viral copies for the lymphoid tissues was considered an indicator of the degree of AMDV infection. The results indicate more-intensive replication of AMDV in the lymph nodes, where the pathogen reached 10^3^copies in the experimental group, while the viral load in the spleen was one order of magnitude lower. A similar relationship was noted in the control group. Irrespective of the tissue examined, the number of viral copies decreased by one order of magnitude following supplementation with methisoprinol.

### Breeding parameters

**Fig. 2:**
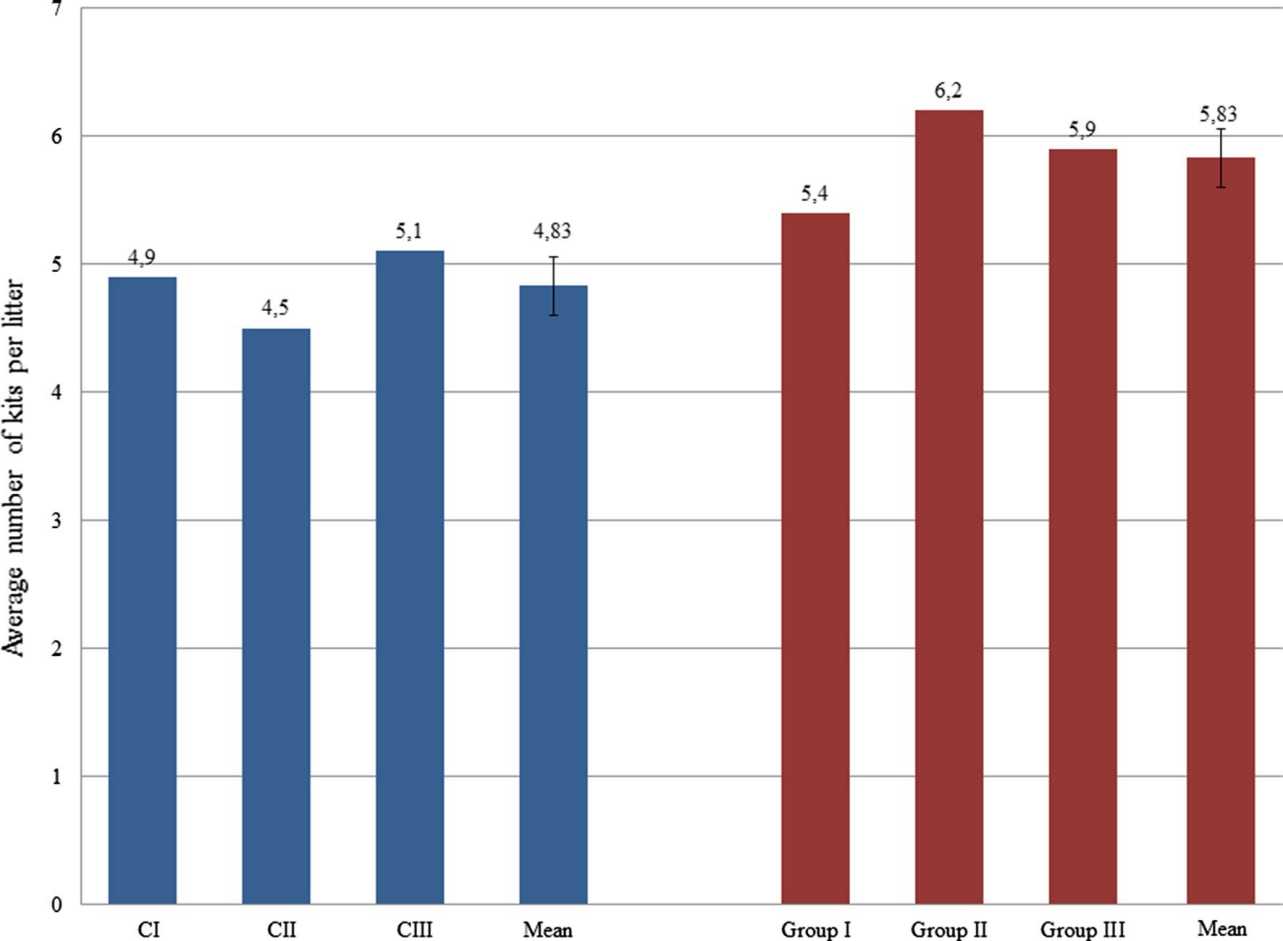
Average number of kits per litter 10 days after whelping; blue, control group; red, experimental group (C1 and group 1 – control and experimental animals with antibody titre designated as +, C2 and group 2 – control and experimental animals with antibody titre designated as ++, C3 and group 3 – control and experimental animals with antibody titre designated as +++)

Breeding parameters were evaluated ten days after whelping (Fig. 2). In the control groups, 78% of 50 normally mated females whelped in group C1, 80% in group C2, and 82% in group C3. The average number of kits per litter in control groups C1, C2 and C3 was 4.9, 4.5 and 5.1 (average 4.83 ± 0.2). In the experimental groups, also numbering 50 females each, 86% of females whelped in group 1, 94% in group 2 and 90% in group 3. The average number of kits per litter was 5.4 in group 1, 6.2 in group 2, and 5.9 in group 3 (average 5.83 ± 0.26). On October 30, i.e. two weeks before slaughter, the total mean number of kits per litter was 4.38 ± 0.31 in the experimental groups of mink and 3.02 ± 0.64 in the control groups. No statistical differences were found between litter size categories. In the experimental groups, the body weight of the young mink before slaughter averaged 1,896.67 g and was 12% higher than in the controls (1,671.67 g – statistically significant difference at *p* = 0.05).

### Molecular characterization of AMDV isolates

The presence of AMDV genetic material was confirmed in both the experimental and control groups. PCR yielded a positive result with both primer pairs. After sequencing, a 1,106-bp fragment of the sequence encoding the structural protein VP2 was obtained. Bioinformatic analysis showed eight variants among the virus isolates, with a high degree of similarity exceeding 98.5%. Variants of AMDV isolates were distinguished on the basis of 16 SNP polymorphisms. A significant difference was found in the nucleotide sequence between the isolates and the Utah, AMDV-G and Pullman reference strains. The isolates were most similar to the Utah 1-kit strain (96% similarity), followed by the nonpathogenic AMDV-G strain (93%), while the lowest similarity was noted in relation to the Pullman strain (91.5%).

## Discussion

Constant and long-term exposure to AMDV can lead to the development of mechanisms that reduce its harmful effects [11]. Attempts have been made at selection of mink for tolerance to the virus in order to obtain individuals with increased resistance to AMDV infection in subsequent generations [12]. Selection is a long-term process, and thus far no markers indicating increased resistance to AMDV have been detected. Therefore, there is a need for alternative methods to counteract the harmful effect of the pathogen on reproductive parameters in mink.

The present study showed that methisoprinol mitigated the effects of infection with Aleutian disease virus. Differences in the number of copies of viral DNA in the spleen and lymph nodes of the mink in the experimental groups indicate a significantly lower rate of AMDV replication (fewer virus copies) in the lymphoid tissues of mink that had received 20% methisoprinol than in the control groups. In the case of the spleen, the replication rate of the virus was higher in young mink than in the females of the foundation stock, while in the lymph nodes the trend was reversed, which may indicate primary replication of the pathogen in the spleen and gradual colonization of the lymphatic system as the animal developed. The preparation exhibits direct antiviral activity through its inhibitory effect on DNA replication and RNA synthesis in the pathogen [5, 13]. An important property of methisoprinol in the case of infection with AMDV is the fact that it induced an increase in phagocyte activity in the spleen [8], the organ with the highest rate of replication of the pathogen, in addition to a normalizing effect on lymphocyte proliferation, inhibiting the harmful effects of hypergammaglobulinaemia [14]. A study by Ahmed et al. [15] demonstrated that a formulation based on inosine acedoben dimepranol caused an increase in the NK cell fraction and thereby enhanced the body’s immune response against viruses, including in immunocompromised individuals. The involvement of NK cells in the response to viral infections has been demonstrated in infections with influenza virus, hepatitis C virus and cytomegalovirus (CMV) [16]. The immunostimulant also strengthens the immune response by increasing such phagocytic parameters as respiratory burst activity (RBA) and potential killing activity (PKA) [7, 17]. Beran et al. [18] have confirmed not only the efficacy of inosine pranobex in the treatment of viral respiratory infections but also its safety for the individuals taking it.

The effects of the immunostimulant have also been confirmed in research conducted by Stenzel et al. [9], who showed that methisoprinol at a dose of 200 mg/kg body weight, administered after infection, inhibited the replication of pigeon paramyxovirus type 1, which infects pigeons. The researchers suggested that the course of the disease was mitigated, despite the presence of the infectious agent, through inhibition of its replication and a reduction in the number of viral copies in the infected tissue; however, RT-PCR did not conclusively verify this hypothesis. In our study, the viral load in both the spleens and the lymph nodes of the mink in the experimental group was one order of magnitude lower than in the control group. The effect of reduced virus replication and the immunostimulatory effect of the drug counteracted the effects of infection, which was manifested by the larger litter size and greater weight of animals from the experimental group. A favourable influence of Isoprinosine on reproduction has also been observed by Humelt et al. [19], who confirmed that immunostimulation of pregnant mares may reduce losses caused by equine herpesvirus 1 (EHV-1). Immunostimulation may also have an impact on other economically important traits. Piech and Brodzki [20] investigated the influence of immunostimulation with Isoprinosine on the hygienic quality of milk from cows with mastitis. The results indicated that supplementation with the preparation improves hygienic parameters of milk and has a beneficial therapeutic effect on cows with mastitis. Procajło et. al. [21] examined the effects of immunostimulation coupled with vaccination against mycoplasmal pneumonia of swine. Administration of methisoprinol 48 h before vaccination resulted in a beneficial effect on health status and production parameters, such as meatiness.

The cost of the treatment may be a factor limiting common use of methisoprinol in prophylaxis of Aleutian disease. Treatment of one animal for three months consumes nearly 50% of the profits from a single fur. However, taking into account the larger litter size obtained in the experimental groups (one more kit per litter than in the control groups), the cost of supplementation for a single female amounts to less than 25% of the income from a single fur. Therefore, the additional income from the larger litter size may compensate for the cost of the supplementation.

Early and specific diagnosis plays a key role in inhibiting the spread of AMDV. Our research has confirmed the high effectiveness of molecular methods based on both PCR and qPCR, which have also been used by other researchers to diagnose Aleutian disease [22–25]. Techniques based on amplification of nucleic acids are increasingly used to supplement serological tests, making it possible to eliminate infected animals from infected farms. In addition to diagnostics, molecular methods are used to assess the molecular epidemiology of the aetiological agent in a given area [2, 3, 26]. Our research, based on polymorphism in the sequence encoding the VP2 protein, showed eight closely related variants of the pathogen. High variability on a single farm has been confirmed by previous analyses conducted in Poland [27], as well as results reported by Canuti et al. [28], who observed considerable variation in the pathogen resulting from the high stocking density on the farm. Polymorphism in the nucleotide sequence of the VP2 protein of the virus may translate into changes in its structure and functionality and thus result in a varied immune response in the host. Hence, in our study, differences in antibody titres in the individual experimental and control groups, determined by the ELISA assay, may have been due to differences in the immune status of the infected mink and the amount of time that had passed since they became infected, as well as the properties of the infecting strain.

In this study, we investigated the effect of a 20% methisoprinol solution on selected breeding parameters of mink infected with Aleutian disease virus, and confirmed its effectiveness against infection with this virus. Given the lack of effective treatment for Aleutian disease and its high incidence, immunostimulation may be a solution for minimizing the harmful effect of the disease on breeding efficiency on farms with persistent AMDV infection. Use of the preparation in mink when they are still young may improve production parameters.

## Electronic supplementary material

Below is the link to the electronic supplementary material.

**Supplementary material 1** Graphical presentation of designed experiment (TIFF 67 kb)
