# Supplementary figures and images for: Breeding parameters on a mink farm infected with Aleutian mink disease virus following the use of methisoprinol
Source: Arch Virol. 2019 Aug 19;164(11):2691–8. doi: 10.1007/s00705-019-04375-x (PMC6768903; doi:10.1007/s00705-019-04375-x)

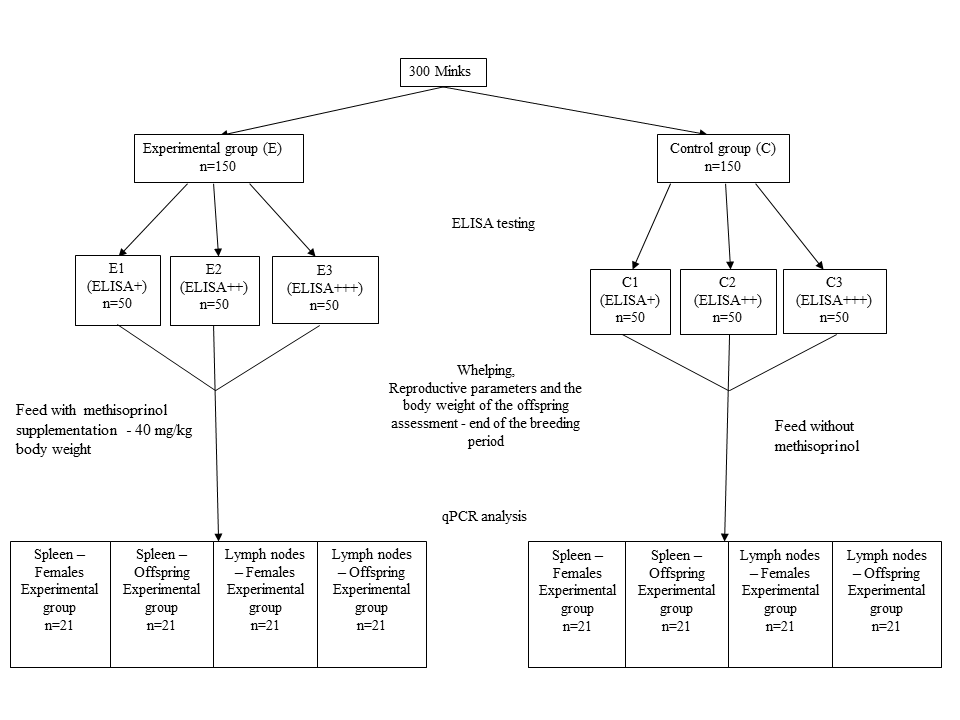

Supplement: Supplementary file 1 — Supplementary material 1 Graphical presentation of designed experiment (TIFF 67 kb) [file 705_2019_4375_MOESM1_ESM.tif]
